# Diagnostic Accuracy of FIB-4 Index and FIB-3 Index for Advanced Fibrosis in Chronic Liver Disease and Age-Specific Thresholds

**DOI:** 10.3390/jcm14238473

**Published:** 2025-11-28

**Authors:** Shohei Kimura, Nobuharu Tamaki, Mayu Higuchi, Takuya Shima, Mina Taguchi, Yudai Yamazaki, Risa Seike, Naoki Uchihara, Yuki Tanaka, Ryohei Kobayashi, Junko Yagita, Yuka Kasano, Yasuyuki Komiyama, Kenta Takaura, Hitomi Takada, Shohei Tanaka, Chiaki Maeyashiki, Yutaka Yasui, Hiroyuki Nakanishi, Kaoru Tsuchiya, Namiki Izumi, Masayuki Kurosaki

**Affiliations:** Department of Gastroenterology and Hepatology, Musashino Red Cross Hospital, 1-26-1 Kyonan-cho, Musashino-shi, Tokyo 180-8610, Japan; sho.12.kim@gmail.com (S.K.); takadahi0107@gmail.com (H.T.);

**Keywords:** non-invasive assessment of liver fibrosis, MR elastography, FIB-4 index, FIB-3 index

## Abstract

**Background and Aims:** Given the high prevalence of chronic liver diseases, non-invasive assessment of liver fibrosis is critical for screening and management of these conditions. While the FIB-4 index is widely used, its age-dependent nature may reduce specificity in older adults. The FIB-3 index, which excludes age, has been proposed as an alternative. This study aimed to compare the diagnostic performance of FIB-3 and FIB-4 for detecting advanced fibrosis across age groups using magnetic resonance elastography (MRE) as the reference standard. **Methods:** We retrospectively analyzed 3424 patients aged ≥30 years with chronic liver disease who underwent MRE. Advanced fibrosis was defined as liver stiffness ≥ 3.62 kPa. The cutoff values and diagnostic performance of the FIB-4 and FIB-3 indices for advanced fibrosis were evaluated within subgroups stratified by age quartiles (Q1–Q4), and the areas under the ROC curves (AUROCs) were compared using DeLong’s test. **Results:** Both FIB-3 and FIB-4 indices demonstrated comparable diagnostic accuracy for advanced fibrosis (AUROC 0.82 (95% CI: 0.81–0.83) for both; *p* = 0.70). In age-stratified analysis, the cutoff values for the FIB-4 index increased with increasing age (Q1: 1.72, Q2: 2.90, Q3: 3.66, Q4: 4.20), reflecting its age-dependent formula. In contrast, the FIB-3 index exhibited relatively minor variation in cutoff values across quartiles (Q1: 2.32, Q2: 3.52, Q3: 3.74, Q4: 3.14). **Conclusions:** FIB-3 index offers similar diagnostic accuracy to FIB-4 index while demonstrating more consistent performance across age groups. These findings provide evidence that FIB-3 index may be a useful alternative, particularly in older populations where FIB-4 index interpretation may be confounded by age.

## 1. Introduction

Liver fibrosis plays a central role in the progression of chronic liver disease, influencing the development of liver-related complications, hepatocellular carcinoma, and overall prognosis [1,2,3,4,5]. Accurate fibrosis staging is therefore essential for effective risk stratification and clinical management.

While liver biopsy remains the gold standard, its invasiveness, procedural risks (e.g., bleeding, pain, infection), and associated costs limit its use in routine practice [6,7,8]. Consequently, non-invasive methods such as magnetic resonance elastography (MRE) have been increasingly adopted [9,10,11,12]. MRE measures liver stiffness by detecting mechanical vibrations using MRI and has shown a strong correlation with histological fibrosis stages [13,14]. MRE is a highly accurate and reproducible technique that has been widely implemented in clinical practice and adopted in clinical trials as a non-invasive alternative to liver biopsy for the assessment of liver fibrosis [15,16,17].

Given the high prevalence of chronic liver diseases—including steatotic liver disease (SLD) and viral hepatitis—in the general population, effective screening tools are essential for identifying individuals with advanced fibrosis [18,19]. The FIB-4 index, which incorporates age, AST, ALT, and platelet count, is widely used for this purpose and is recommended by various clinical guidelines as a first-line screening tool [20,21,22,23].

However, recent studies have emphasized the limitations of FIB-4 in older adults, suggesting that the inclusion of age in the FIB-4 formula may lead to elevated scores and a higher rate of false positives in older individuals [24,25,26]. To address this limitation, Kariyama et al. first proposed the FIB-3 index in 2022, a simplified model that excludes age [27]. In a study of 606 patients aged ≥60 years, the overall classification accuracy for predicting F3 or F4 fibrosis was 0.529 (95% CI, 0.488–0.569) for the FIB-4 index and 0.645 (95% CI, 0.606–0.683) for the FIB-3 index, supporting the superior performance of the FIB-3 index in older adults [28].

Nevertheless, most prior studies on this topic were single-center or small-cohort investigations, and the diagnostic performance of the FIB-3 index in broader populations remains to be clarified. Further studies are warranted to assess its generalizability and clinical utility across diverse patient groups.

In this study, we aimed to compare the diagnostic performance of the FIB-3 and FIB-4 indices in predicting advanced liver fibrosis, using MRE as the reference standard, with particular attention to their performance across different age groups.

## 2. Methods

### 2.1. Study Protocol

This retrospective cohort study was conducted at Musashino Red Cross Hospital, a tertiary referral center for liver disease in Tokyo, Japan. A total of 3529 consecutive patients with chronic liver disease who underwent magnetic resonance elastography (MRE) between January 2015 and October 2024 were initially considered for inclusion. Patients with missing blood test data (*n* = 43), those under 30 years of age (*n* = 54) and those with failed MRE acquisition due to technical failure or poor image quality (*n* = 8). Cases of acute hepatitis were excluded, because MRE is not routinely performed in this condition. The final study cohort consisted of 3424 patients.

### 2.2. MRE Assessment

MRE was performed using a Signa HDxt 1.5T MRI system (GE Medical Systems, Waukesha, WI, USA) equipped with MR Touch (GE Healthcare) [29]. Shear waves were generated by applying a 60 Hz external vibration through a passive driver positioned laterally to the xiphoid process. Cross-sectional elastography images were acquired using gradient echo sequences, and liver stiffness maps were constructed from the resulting wave propagation data.

Regions of interest (ROIs) were placed in the right hepatic lobe, carefully avoiding the liver surface, edges, gallbladder, blood vessels, bile ducts, tumors, and imaging artifacts. The mean liver stiffness value was calculated from three circular ROIs placed on separate image slices. For this study, a liver stiffness value of ≥3.62 kPa was defined as indicative of advanced fibrosis (≥F3), in accordance with prior validation studies.

### 2.3. Clinical and Laboratory Data

Laboratory parameters—including aspartate aminotransferase (AST), alanine aminotransferase (ALT), and platelet count (PLT)—were obtained from routine clinical blood tests performed within 1 month of the MRE. The median (minimum–maximum) values of these parameters were summarized for the entire cohort and each age quartile in Table 1.

The FIB-4 index was calculated using the following formula:

FIB-4 index = (Age × AST [U/L])/(PLT [10^9^/L] × √ALT [U/L])

The FIB-3 index was calculated as:

FIB-3 index = 5 × ln(AST [U/L]) − 2 × ln(ALT [U/L]) − 0.18 × PLT [10^4^/μL] − 5

### 2.4. Primary Endpoint

The primary objective of this study was to compare the diagnostic performance of the FIB-4 and FIB-3 indices in identifying advanced fibrosis (≥F3), as defined by MRE. Diagnostic accuracy, sensitivity, and specificity were evaluated in the overall cohort and within age-stratified subgroups.

### 2.5. Statistical Analysis

Receiver operating characteristic (ROC) curve analysis was performed to assess the predictive performance of each index. Optimal cutoff values were determined using the Youden index, and corresponding sensitivity and specificity were calculated.

To evaluate age-related differences, diagnostic performance was further analyzed across quartiles of age:

Q1: Age < 59, Q2: 59 ≤ Age < 69, Q3: 69 ≤ Age < 75, Q4: Age ≥ 75

The areas under the ROC curves (AUROCs) for FIB-4 and FIB-3 indices were compared using DeLong’s test. All statistical analyses were conducted using EZR (Easy R), a graphical user interface for R.

## 3. Results

### 3.1. Patient Characteristics

A total of 3424 patients were included in the final analysis. The median age was 68 years (minimum–maximum: 59–75), and 48.5% (*n* = 1661) were male. MRE revealed a median liver stiffness of 3.56 kPa (1.37–19.4), with 48.7% of patients (*n* = 1669) exceeding the threshold of 3.62 kPa, indicative of advanced fibrosis (≥F3). The median FIB-4 index was 2.69 (0.39–27.8), while the median FIB-3 index was 2.78 (−4.49–12.5), indicating a broad distribution of fibrosis severity in the cohort.

The underlying etiologies of liver disease were as follows: hepatitis C virus (HCV) in 57.6% (*n* = 1971), hepatitis B virus (HBV) in 11.9% (*n* = 408), metabolic dysfunction-associated steatotic liver disease or metabolic dysfunction-associated steatohepatitis (MASLD/MASH) in 6.1% (*n* = 210), alcohol-related liver disease in 4.7% (*n* = 161), and other causes in 19.7% (*n* = 674). Among patients with hepatitis C virus (HCV) infection, 1252 had achieved sustained virologic response (SVR), while 719 were treatment-naïve at the time of MRE.

### 3.2. Diagnostic Performance of the FIB-4 and FIB-3 Indices for Advanced Fibrosis

**Figure 1:**
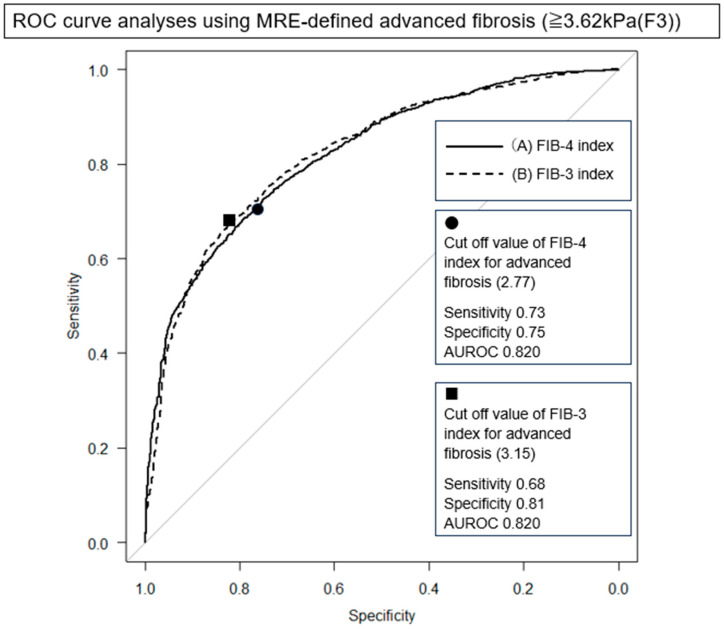
ROC curve analyses of FIB-4 index and FIB-3 index for advanced fibrosis. (A) FIB-4 index, and (B) FIB-3 index. Advanced fibrosis is defined MRE of ≥3.62kPa. The diagonal gray line represents the line of no discrimination.

To assess the diagnostic ability of the FIB-4 and FIB-3 indices to detect advanced fibrosis (defined as MRE ≥ 3.62 kPa), ROC curve analysis was performed (Figure 1). Both FIB-4 (AUROC 0.82) and FIB-3 (AUROC 0.82) indices demonstrated comparable diagnostic performance. At the optimal cutoff value of 2.77, FIB-4 index achieved a sensitivity of 72.7%, specificity of 75.0%, and overall accuracy of 73.9%. The FIB-3 index, using an optimal cutoff of 3.15, showed a sensitivity of 68.0%, specificity of 81.3%, and accuracy of 74.9%. The difference in diagnostic performance between the two indices was not statistically significant (*p* = 0.70).

When stratified by gender, the AUROCs of FIB-3 and FIB-4 were similar in both females (0.84 vs. 0.84, *p* = 0.69) and males (0.81 vs. 0.81, *p* = 0.57), indicating no significant difference between the indices in either sex.

### 3.3. Comparison of Diagnostic Performance Across Age Quartiles

Further analysis was conducted by stratifying patients into age quartiles and evaluating the performance of each index within these subgroups (Table 2). The cutoff values for the FIB-4 index increased with increasing age (Q1: 1.72, Q2: 2.90, Q3: 3.66, Q4: 4.20), reflecting its age-dependent formula. In contrast, the FIB-3 index exhibited relatively minor variation in cutoff values across quartiles (Q1: 2.32, Q2: 3.52, Q3: 3.74, Q4: 3.14). When evaluated using a single cutoff value (FIB-4 index: 2.77; FIB-3 index: 3.15), the accuracies across quartiles were as follows: FIB-4 index, 81.0% (Q1), 73.4% (Q2), 68.7% (Q3), and 71.7% (Q4); FIB-3 index, 80.9% (Q1), 75.8% (Q2), 68.8% (Q3), and 73.6% (Q4). No statistically significant differences in AUROC were observed between FIB-4 and FIB-3 indices within any age group.

**Figure 2:**
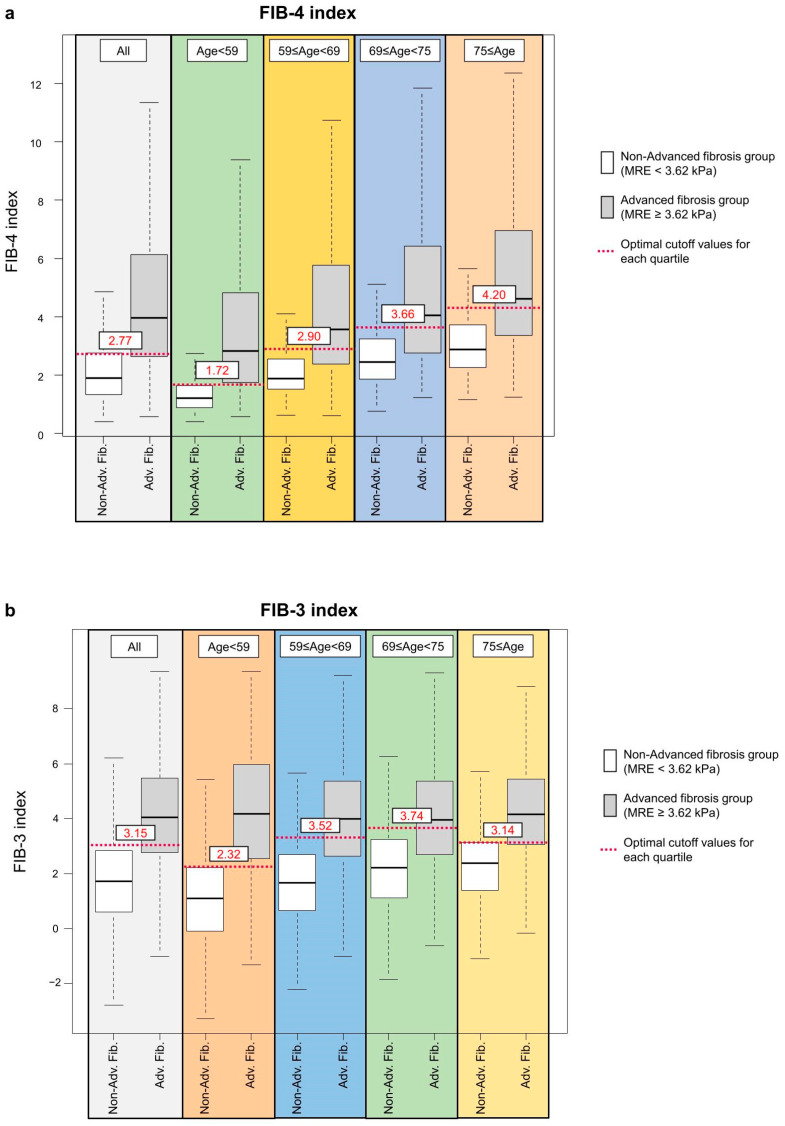
(**a**) Effect of age on the FIB-4 index for detecting advanced fibrosis. (**b**) Effect of age on the FIB-3 index for detecting advanced fibrosis. Patients are stratified by age quartiles and fibrosis stage. The bottom and top of each box represent the 25th and 75th percentiles, representing the interquartile range. The line through the box indicates the median value. The whiskers extend to the most extreme values within [Q1 − 1.5 × IQR] and [Q3 + 1.5 × IQR]; observations outside this range are not plotted. The white boxes represent non-advanced fibrosis, and the gray boxes represent advanced fibrosis. The red dashed line indicates the optimal cutoff value for stratifying non-advanced and advanced fibrosis.

Box-and-whisker plots (Figure 2) revealed greater stability in the distribution of the FIB-3 index across age quartiles. Specifically, the FIB-3 index showed less variation in median values and interquartile ranges compared to the FIB-4 index.

## 4. Discussion

### 4.1. Main Findings

In this study, we compared the diagnostic performance of the FIB-3 and FIB-4 indices for predicting advanced liver fibrosis (≥F3), using magnetic resonance elastography (MRE) as the reference standard. Both indices demonstrated comparable diagnostic accuracy, with no statistically significant difference between them. These findings confirm that FIB-3 index performs equivalently to FIB-4 index in the non-invasive assessment of liver fibrosis.

In age-stratified analyses, the FIB-3 index maintained stable performance across all age quartiles, with modest variation in cutoff thresholds. In contrast, the optimal cutoff values for FIB-4 index increased with age, reflecting its age-dependent formula. These results provide evidence that FIB-3 index offers more consistent performance across different age groups.

### 4.2. Comparison with Published Literature

The FIB-4 index has been widely used and validated as a non-invasive marker for liver fibrosis, particularly in patients with viral hepatitis and metabolic liver disease [22]. However, its age-dependent nature leads to diagnostic overestimation in older individuals [24]. Age-adjusted cutoff values have been proposed to address this limitation, but applying them in large populations remains challenging.

The FIB-3 index, which excludes age and incorporates AST, ALT, and platelet count, provides a simpler alternative [27]. Although fewer studies have evaluated FIB-3 index, emerging evidence demonstrates its acceptable diagnostic accuracy with reduced age-related variability [28,30]. Our results align with these observations, showing that the FIB-3 index achieves diagnostic performance comparable to the FIB-4 index while exhibiting less fluctuation in cutoff values across age groups.

### 4.3. Strengths and Limitations

The strengths of this study include a large sample size, non-invasive reference standard, and detailed stratified analyses by age. These features allowed a comprehensive assessment of overall diagnostic performance and age-related variability.

However, several limitations should be considered. This was a retrospective, single-center study, which may limit generalizability. Histological confirmation was not available in most cases. Although the FIB-3 index showed promising results, further validation in diverse and independent cohorts is needed before broad clinical adoption, and additional studies should clarify its robustness and clinical utility across different clinical settings.

### 4.4. Future Implications

Our findings suggest that the FIB-3 index serves as a practical and age-stable alternative to the FIB-4 index in the non-invasive evaluation of liver fibrosis. Given its comparable performance and reduced variability across age groups, the FIB-3 index could be particularly useful for population-based screening in the general community, where age diversity is substantial.

Future studies should validate its effectiveness in primary care and community screening settings, and assess its ability to identify individuals at risk for advanced fibrosis who may benefit from early intervention.

## 5. Conclusions

The FIB-3 index demonstrated diagnostic performance comparable to the FIB-4 index for detecting advanced liver fibrosis and showed greater stability across age groups. However, this was a retrospective single-center study without routine histological confirmation, which may limit the strength of our conclusions. Further prospective, multicenter studies with histological validation, as well as evaluation in population-based screening settings, are required to confirm the clinical utility of the FIB-3 index.

## Figures and Tables

**Table 1:** Patients Characteristics in the entire population and in each age stratification.

	All *n* = 3424	Q1: Age < 59 *n* = 885	Q2: 59 ≤ Age < 69 *n* = 892	Q3: 69 ≤ Age < 75 *n* = 799	Q4: Age ≥ 75 *n* = 848
Age	68 (30, 96)	52 (30–58)	65 (59–68)	72 (69–75)	80 (75–96)
Sex (Female/Male)	1763 (51.5%)/1661 (48.5%)	370 (41.8%)/515 (58.2%)	457 (51.2%)/435 (48.8%)	424 (53.1%)/375 (46.9%)	512 (60.4%)/336 (39.6%)
Etiology					
HBV	408 (11.9%)	175 (19.8%)	123 (13.8%)	74 (9.3%)	36 (4.2%)
HCV	1971 (57.6%)	373 (42.1%)	502 (56.3%)	487 (61.0%)	609 (71.8%)
MASLD/MASH	210 (6.1%)	35 (4.0%)	60 (6.7%)	52 (6.5%)	63 (7.4%)
ALD	161 (4.7%)	49 (5.5%)	55 (6.2%)	42 (5.3%)	15 (1.8%)
Others	674 (19.7%)	253 (28.6%)	152 (17.0%)	144 (18.0%)	125 (14.7%)
MRE (kPa)	3.56 (1.37–19.4)	2.84 (1.44–19.0)	3.64 (1.37–19.4)	3.71 (1.60–14.4)	4.22 (1.67–13.7)
≥3.62 kPa	1669 (48.7%)	282(31.9%)	449 (50.3%)	420 (52.6%)	518 (61.1%)
Platelets (×10^4^/μL)	16.2 (2.6–53)	19.5 (2.6–44.8)	16.0 (2.7–53)	15.2 (2.8–41)	14.6 (2.9–40)
AST (U/L)	30 (9–266)	32 (10–266)	29 (9–182)	30 (11–211)	31 (11–254)
ALT (U/L)	25 (4–197)	33 (6–193)	24 (4–197)	24 (5–193)	23 (6–190)
GGT (IU/L)	29 (6–1439)	40 (8–1439)	30 (6–1156)	29 (8–658)	26 (7–740)
Albumin (g/dL)	4.2 (2.1–5.6)	4.3 (2.2–5.4)	4.2 (2.3–5.6)	4.1 (2.1–5.2)	4.0 (2.3–5.1)
Bilirubin (mg/dL)	0.7 (0.2–6.9)	0.7 (0.2–6.9)	0.7 (0.2–5.8)	0.7 (0.2–2.6)	0.7 (0.2–3.2)
FIB-4 index	2.69 (0.39–27.8)	1.45 (0.39–24.8)	2.50 (0.61–27.8)	3.04 (0.77–20.5)	3.75 (1.14–24.9)
FIB-3 index	2.78 (−4.49–12.5)	1.79 (−4.49–12.5)	2.68 (−4.61–10.3)	3.01 (−3.10–10.9)	3.22 (−1.86–10.2)

Data are expressed as number (%) of patients or median (minimum–maximum). HBV: hepatitis B virus, HCV: hepatitis C virus, ALD: alcoholic liver disease.

**Table 2:** Comparison of FIB-4 index and FIB-3 index between age quartiles.

		Cut Off	Sensitivity	Specificity	AUROC
All Ages	FIB-4 index	2.77	0.73	0.75	0.82
	FIB-3 index	3.15	0.68	0.81	0.82
1st QR Age < 59	FIB-4 index	1.72	0.76	0.80	0.85
	FIB-3 index	2.32	0.80	0.77	0.86
2nd QR 59 ≤ Age < 69	FIB-4 index	2.90	0.64	0.86	0.82
	FIB-3 index	3.52	0.62	0.90	0.82
3rd QR 69 ≤ Age < 75	FIB-4 index	3.66	0.57	0.86	0.77
	FIB-3 index	3.74	0.55	0.85	0.76
4th QR 75 ≤ Age	FIB-4 index	4.20	0.56	0.88	0.79
	FIB-3 index	3.14	0.73	0.75	0.79

## Data Availability

All data relevant to the study are included in the article. Data details can be provided upon request to credible investigators on verification for patient confidentiality.
